# Case Report: A Rare Presentation of NSAID-Induced Secondary Membranous Nephropathy in a Pediatric Patient

**DOI:** 10.3389/fped.2021.670575

**Published:** 2021-04-23

**Authors:** Siddharth Shah, M. Asope Elder, Jessica Hata

**Affiliations:** ^1^Department of Pediatrics, Norton Children's and University of Louisville, Louisville, KY, United States; ^2^Department of Pathology, Norton Children's and University of Louisville, Louisville, KY, United States

**Keywords:** NSAID, membranous nephropathy, proteinuria, nephrotic syndrome, ACE inhibitor

## Abstract

**Background:** Membranous nephropathy (MN) is a common cause of nephrotic syndrome in adults, but it is responsible for <5% of nephrotic syndrome cases in children. MN has primary and secondary forms. Secondary MN is caused by viral infections, autoimmune diseases like lupus, or drugs. Non-steroid anti-inflammatory drug (NSAID)-induced secondary MN is rarely described in the pediatric population. Thus, the clinical presentation and time to recovery are vastly unknown in the pediatric subgroup.

**Clinical Presentation:** We report a case of a 15-year-old female who presented with acute onset of nephrotic range proteinuria, significant hypoalbuminemia, hyperlipidemia, and lower extremity edema related to the presence of nephrotic syndrome. She had a history of ibuprofen use periodically for 6 months before presentation because of menstrual cramps and intermittent lower abdominal pain. After the presentation, we performed a renal biopsy that reported stage 1–2 MN, likely secondary. The phospholipase A2 receptor (PLA2R) antibody on the blood test and PLA2R immune stain on the renal biopsy sample were negative. We performed a comprehensive evaluation of the viral and immune causes of secondary MN, which was non-revealing. She had stopped ibuprofen use subsequent to the initial presentation. She was prescribed ACE inhibitor therapy. After 6 months of ACE inhibitor treatment, the proteinuria had resolved.

**Conclusion:** Proteinuria can last for several weeks when NSAID induces secondary MN and nephrotic syndrome. With the widespread use of NSAIDs prevalent in the pediatric community, further studies are needed to evaluate and study the role of NSAIDs in this condition.

## Introduction

Membranous nephropathy (MN) is a rare cause of nephrotic syndrome in the pediatric population and contributes to <5% of childhood nephrotic syndrome cases (1). Primary MN is a renal-specific autoimmune glomerular disease characterized by proteinuria, primarily mediated by antibodies against phospholipase A2 receptor (PLA2R). It is characterized by specific histological findings on renal biopsy, such as a pathognomic “spike pattern” of the glomerular basement membrane on light microscopy (LM); positive PLA2R immunostaining, IgG and complement C3 distributed in a delicate granular pattern distributed across a subepithelial portion of glomeruli on immunofluorescence microscopy (IFM); and electron-dense deposits confined to the subepithelial space on electron microscopy (EM), with the absence of mesangial deposits (2, 3). Secondary MN has previously been shown to be caused by viral infections (e.g., Hepatitis B, Hepatitis C, or HIV), autoimmune conditions (e.g., lupus), drugs (e.g., penicillamine, gold, or NSAIDs), hematological malignancies, and solid organ tumors (1, 2). The histological findings of secondary MN can vary depending on the cause, and PLA2R immunostaining is negative (2). Primary MN is a more common form of MN in adults in the literature review, comprising ~80% of total MN cases, but secondary MN may be more common in children (1). Due to the rarity of secondary MN in the pediatric population and the lack of controlled studies, there are no reliable treatment guidelines or predictions of outcomes for this condition.

## Patient Information

We report the case of a 15-year-old female (weight 68.3 kg, BMI of 22.56) who presented with acute onset bilateral lower extremity edema and fatigue. She had gained nine pounds of weight at presentation compared to her previous weight 1 month earlier. She denied using any medications, except she said she had been taking ibuprofen at high doses (1,600–2,400 mg/day) intermittently for the past 6 months because of menstrual cramps and intermittent lower abdominal pain. She rejected any illicit substance use and was living at home with her parents and sibling.

## Clinical Findings

The comprehensive metabolic panel suggested the presence of hyponatremia, with a serum sodium level of 132 meq/L (reference range: 135–145 meq/L), and significant hypoalbuminemia, with a serum albumin level of 2.4 g/dL (reference range: 3.6–5.1 g/dL). Renal function was normal, based on blood urea nitrogen (BUN) of 11 mg/dL (reference range: 4–20 mg/dL) and serum creatinine of 0.5 mg/dL (reference range: 0.5–1 mg/dL). The complete blood count was acceptable, with a WBC count of 13.62 × 10^3^/uL, hemoglobin of 12.4 g/dL, and a platelet count of 407 × 10^3^/uL. The serum lipid profile was abnormal, with a total cholesterol level of 366 mg/dL (reference range: <170 mg/dL) and elevated serum LDL of 249 mg/dL (reference range: <110 mg/dL). The urine dipstick showed +3 protein and was negative for blood. The random urine albumin-to-creatinine ratio was high: 5,229 mcg/mg of creatinine (reference normal <30 mcg/mg of creatinine). The renal ultrasound was normal.

## Diagnostic Assessment

A clinical diagnosis of nephrotic syndrome was made based on hypoalbuminemia, hyperlipidemia, and heavy proteinuria. Although minimal change disease (MCD) is a common cause of nephrotic syndrome in pediatric patients, the risk of focal segmental glomerulosclerosis (FSGS) increases when the onset of nephrotic syndrome occurs in late childhood or adolescence (4).

**Figure 1:**
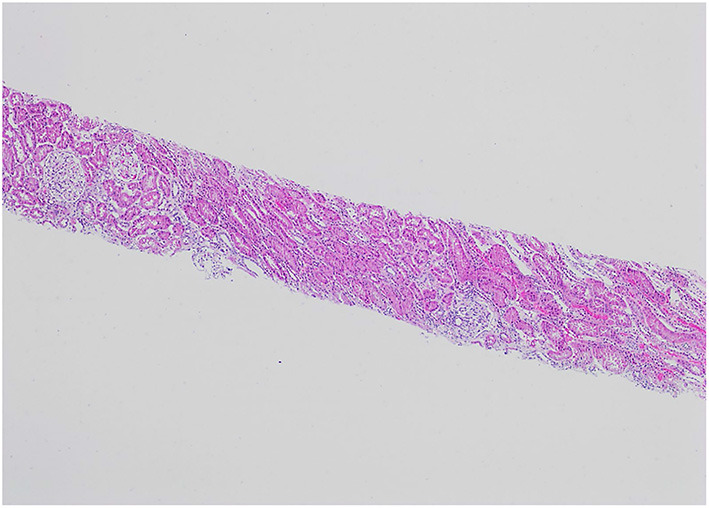
H&E, 4X: The renal biopsy shows glomeruli with normal cellularity and no significant tubular atrophy or interstitial fibrosis.

**Figure 2:**
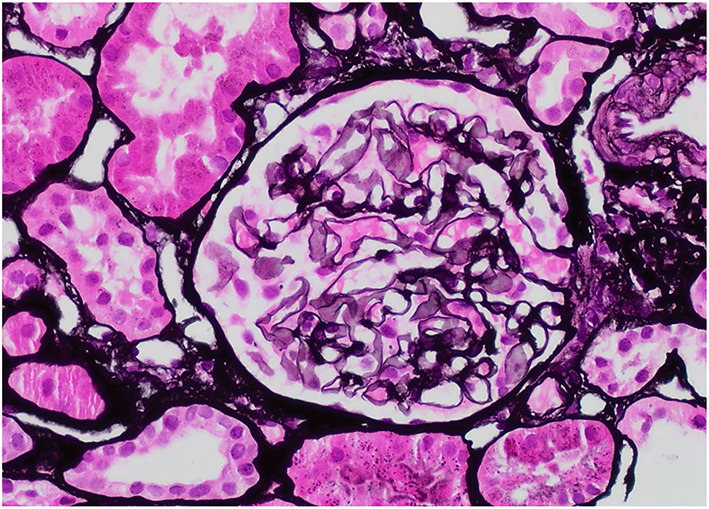
Jones Silver, 40X: The glomerulus lacks well-developed “spikes” or “holes”.

**Figure 3:**
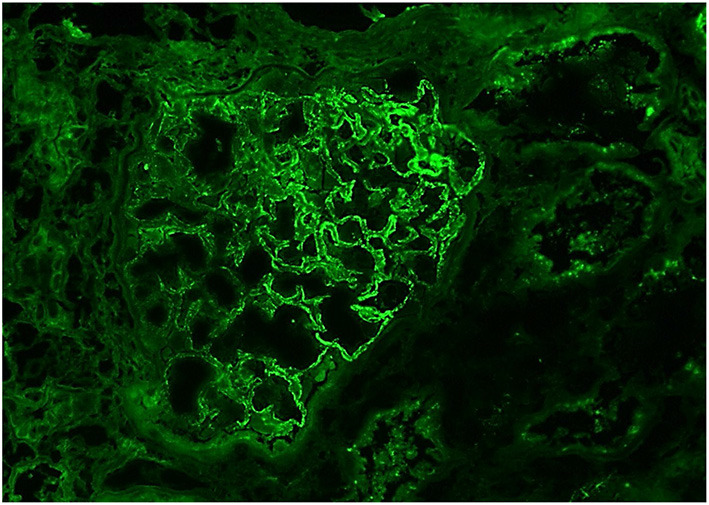
IgG, 40X: The glomerulus demonstrates mesangial and diffuse global, granular capillary loop staining.

**Figure 4:**
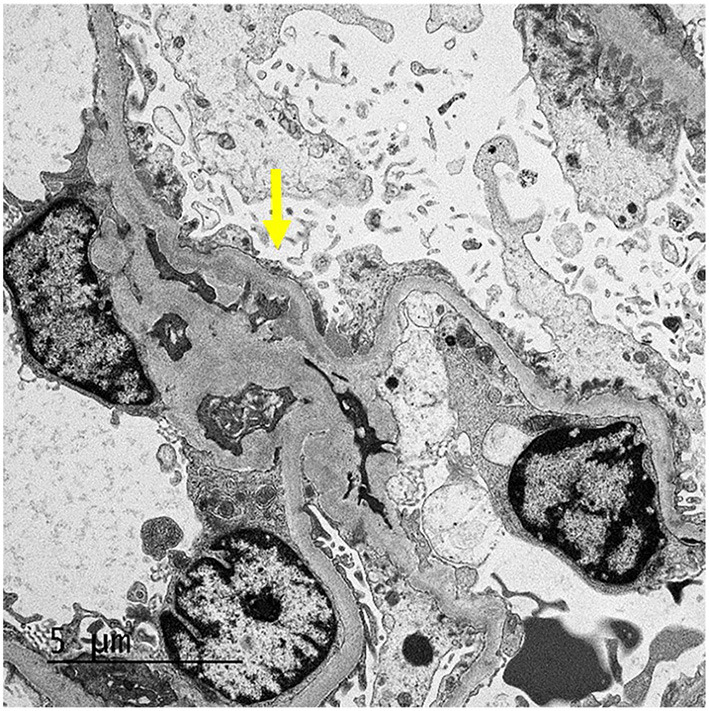
EM: The glomerulus demonstrates mesangial (arrow) and segmental small subepithelial electron-dense deposit.

**Figure 5:**
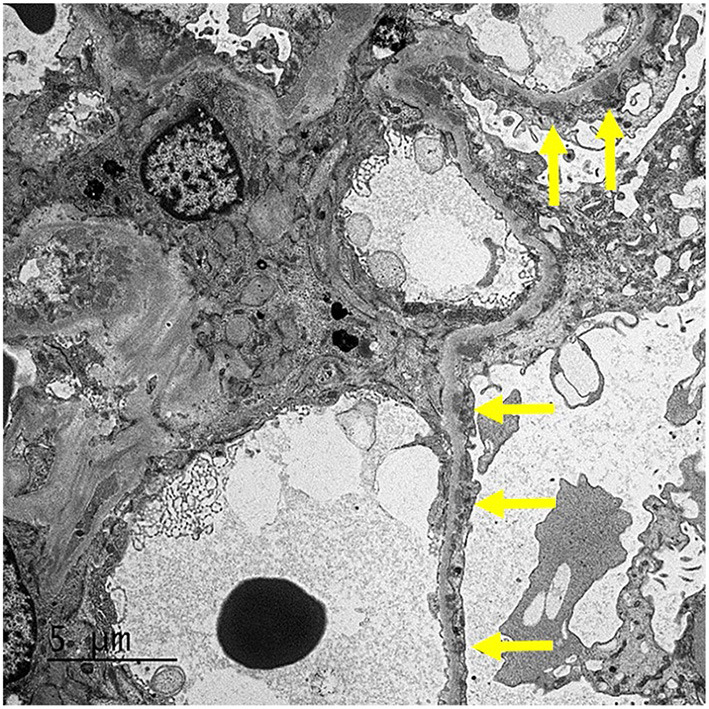
EM: The glomerulus demonstrates mesangial and segmental small subepithelial electron-dense deposit (arrows).

Our patient was 15 years of age at presentation; hence, an early renal biopsy was performed to determine the cause. The glomeruli appeared normal overall on light microscopy (LM), and there were no “spikes” or “holes” in the glomerular basement membrane (Figures 1, 2). There was no significant tubular atrophy, interstitial inflammation or interstitial fibrosis. Even though MN is rare among pediatric patients, primary MN was in the differential given the patient's age. We performed PLA2R immunostain on renal biopsy specimens to differentiate primary and secondary MN as there are differences in treatment in these two conditions. On immunofluorescence microscopy (IFM), the PLA2R immunostain was negative, but there was positive mesangial and glomerular diffuse capillary loop staining for IgG, kappa, and lambda (Figure 3). There was also positive staining for segmental granular mesangial C1q. Further, we saw negative staining for IgA and C3, and there was no full-house pattern on IFM, which made lupus less likely as a plausible diagnosis. Electron microscopy (EM), revealed the presence of mesangial and diffuse subepithelial electron-dense deposits (Figures 4, 5). There were no tubule-reticular bodies (as seen with lupus), and podocytes were effaced on EM. Given the presence of subepithelial electron-dense deposits and diffuse capillary loop staining for IgG, a diagnosis of MN was made (1). However, the classical findings of primary MN, such as a “spike pattern” of the glomerular basement membrane, C3 staining on immunofluorescence, and positive PLA2R staining, were absent, leading to an increased possibility of secondary MN. Additionally, there was an additional presence of mesangial electron-dense deposits and positive C1q staining, which is reported with cases of secondary MN (1, 2). Viral and immune studies were performed to evaluate the causes of secondary MN and found to be non-revealing (Table 1). Based on the above findings and the patient's history of high- dose ibuprofen use, the diagnosis of NSAID-induced secondary MN was made.

**Table 1:** Viral and immunological studies.

**Test**	**Result**	**Reference/normal**
Rheumatoid factor (RF)	<14 IU/ml	<14 IU/ml
Thyroid stimulating hormone (TSH)	3.78 u(iU)/mL	0.470–4.680 u(iU)/mL
Immunoglobulin G (IgG)	488 mg/dL	500–1,590 mg/dL
Antinuclear antibody (ANA) screen	Negative	Negative
Complement C3	217 mg/dL	83–193 mg/dL
Complement C4	43 mg/dL	15–57 mg/dL
Hepatitis panel (Hepatitis A IgM, Hepatitis B surface Ag, Hepatitis B core Ab, Hepatitis C antibody)	Negative	Negative
HIV screen	Negative	Negative
Cytomegalovirus PCR	Negative	Negative
Epstein-Barr virus PCR	Negative	Negative
Serum protein electrophoresis	Normal	
Serum phospholipase A2 receptor antibody (PLA2R)	Negative	Negative

## Therapeutic Assessment

Our patient stopped using NSAIDs after the initial presentation. She was also initially started on steroids (prednisone) because of her presentation with nephrotic syndrome and based on the available pediatric literature review, but they were weaned over 4 weeks because of a lack of improvement of proteinuria and a strong suspicion of secondary MN (1). Because of the lack of data on treatment options for NSAID-induced secondary MN in pediatric patients, we reviewed the available literature on adult patients, where the treatment varies and includes no treatment, ACE inhibitor use, and steroid use (5–7). Apparently, proteinuria can last for several weeks after stopping NSAIDs in NSAID-induced secondary MN (2, 6). We initiated treatment with an ACE inhibitor and monitored the improvement in proteinuria.

## Follow-Up and Outcomes

**Figure 6:**
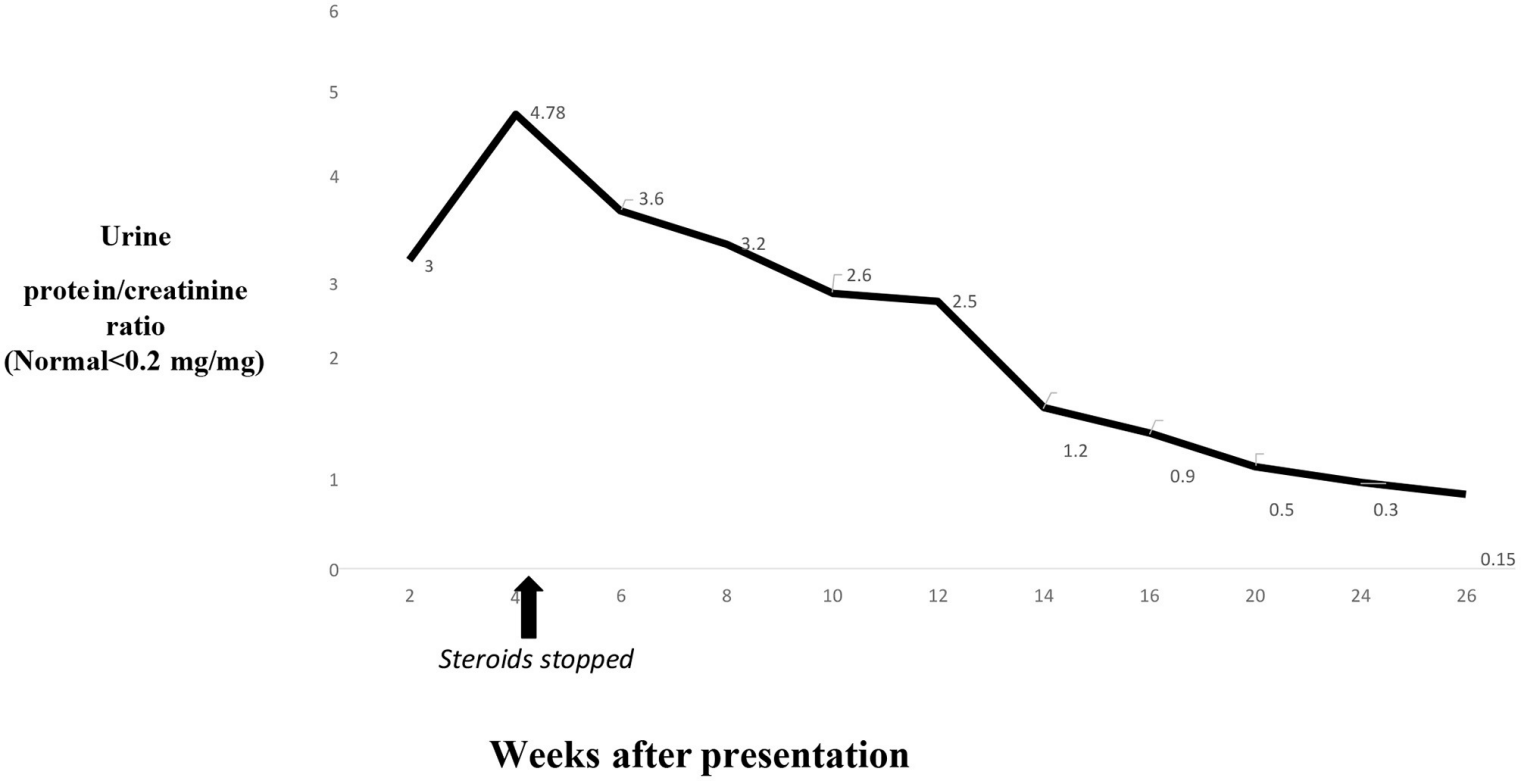
Trends in proteinuria on ACE inhibitor therapy.

The swelling on extremities improved in 6–8 weeks, while the patient's serum albumin level took 4 months to return to the normal range after the initial presentation. The proteinuria took over 6 months to resolve completely (Figure 6). She had dizziness on 10 mg/day lisinopril, and dose had to be reduced to 7.5 mg/day. Lisinopril was stopped 6 months after presentation. She continues to be in remission and has had no episodes of relapse or significant proteinuria 1 year after presentation.

## Discussion

NSAIDs are widely used in the pediatric population to treat pain or fever, and their side effects on kidneys are often not well-recognized. The absence of evident symptoms often leads to a delay in the diagnosis of NSAID-induced kidney disease (8). NSAIDs cause acute kidney injury (AKI) secondary to pre-renal vasoconstriction, acute tubular necrosis (ATN), or acute tubulointerstitial nephritis (ATIN) (8). In hypovolemic states, the production of prostaglandins is upregulated to maintain renal blood flow. NSAIDs inhibit the cyclooxygenase (COX) enzyme, which affects the biosynthesis of prostaglandins, leading to pre-renal vasoconstriction, pre-renal AKI, and ATN in association with hypovolemia (9). It also leads to ATIN and enlargement of kidneys due to an immuno-allergic reaction (8). In terms of nephrotic syndrome, both MCD and MN have been reported with NSAID use (10). MN has been reported with all NSAIDs, including selective COX-2 inhibitors (2, 5). The exact mechanism of NSAID-induced secondary MN is unknown. Different theories have been proposed, including COX inhibition, glomerular deposition of antigens that bind to NSAIDs, or NSAIDs triggering autoimmune reaction against antigens in the glomerular filtration barrier (2, 10).

The duration of NSAID treatment prior to the development of MN is highly variable, from a few weeks to a few months, but there is a rapid development of nephrotic-range proteinuria or presentation with nephrotic syndrome at disease onset (2). Because the symptoms of hypoalbuminemia and edema may be more apparent in NSAID-induced MN, an early renal biopsy is often performed, and this also explains the early stage of MN (stage I or II) often seen on renal biopsies in these patients (2). Our patient also had stage I–II MN on renal biopsy. Since NSAIDs can cause nephrotic syndrome with both MCD and MN presentation, the presence of electron-dense subepithelial deposits and podocyte effacement on EM and the absence of interstitial inflammatory infiltrates on LM are characteristic findings of NSAID-induced MN. In contrast, interstitial infiltrates typically present in NSAID-induced MCD (10). Because of the absence of interstitial infiltrates, renal function can be generally normal in patients with NSAID- induced MN, as also seen in our patient (10). The absence of interstitial infiltrates on renal biopsy could also explain the normal kidney sizes and the lack of enlarged kidneys seen in our patient on renal ultrasound. The lack of a classical “spike” pattern on LM, the absence of PLA2R immune-stain on IFM and negative serum PLA2R antibody screen, and the mesangial presence of deposits along with sub-epithelial deposits on EM led to increased suspicion of secondary MN, as seen in previous reports (1, 2).

A previous retrospective case series published before the availability of PLA2R immune-stain diagnosed the presence of NSAID-induced MN when patients had early-stage I–II MN on renal biopsy; the patients were concurrently taking NSAIDs at the presentation of nephrotic syndrome, and other causes of MN were not detected (6). This series identified 13 adult patients with NSAID-induced MN, and the duration of NSAID use ranged from 1 to 36 months prior to disease onset, the duration of symptoms, such as edema, ranged from 1 to 24 weeks, and treatment ranged from the withdrawal of NSAIDs to further steroid or ACE inhibitor use (6). Interestingly, the time for improvement of proteinuria (as evaluated by 24-h urine protein <1 g) ranged from 9 to 40 weeks after disease presentation among patients who were regularly followed (6). Our patient was treated with an ACE inhibitor and regularly followed, but proteinuria lasted for 26 weeks after presentation. The improvement criteria for proteinuria in our patient were more stringent, as observed by a random urine protein and creatinine ratio <0.2, and this may have also explained the longer duration of proteinuria. Once the patient was in remission, no relapse was observed, in line with previous reports of NSAID-induced MN (2, 6). A randomized controlled trial comparing the anti-proteinuric effects of lisinopril and losartan treatment for 27 patients with primary MN showed a significant reduction in proteinuria in both groups after 12 months of follow-up (11). However, the role of ACE inhibitors in secondary MN and whether they help to decrease the duration of proteinuria or change the natural course of the disease after stopping NSAIDs needs to be further studied.

There are occasional pediatric case reports of drug-induced secondary MN from pencillamine, but the use of such medicines is now decreasing while NSAID use remains prevalent (12). To the best of our knowledge, this report is one of the initial comprehensive case reports on NSAID- induced MN and its outcome in a pediatric patient, as the literature regarding this topic is limited in this population. Our patient had some unique features on renal biopsy that may provide more insight into this challenging condition.

## Conclusion

NSAIDs are widely used in children and adolescents and can lead to side effects on kidneys, which often go unrecognized. The diagnosis of NSAID-induced secondary MN is challenging and requires a proper laboratory work-up and histological testing. Proteinuria can last for several weeks, even after the resolution of edema in this condition. Further studies are needed to better understand the natural history and long-term outcomes of NSAID-induced secondary membranous nephropathy. More awareness is needed regarding the effects of NSAIDs on kidneys, and their careful use would help to decrease the incidence of NSAID-induced nephrotic syndrome in children.

## Data Availability Statement

The original contributions presented in the study are included in the article/supplementary material, further inquiries can be directed to the corresponding author/s.

## Ethics Statement

The case report was reviewed and approved by the Institutional review Board at Louisville, KY, USA. We obtained written informed consent from the parent to publish any potentially identifiable data included in this report.

## Patient Perspective

Note from Father:

Yes, as a father I can say that for me and her mother it was very hard to come to terms with the fact that something wrong had happened just because of a simple over the counter medication. It took us aback and made us nervous.

## Author Contributions

SS was the primary pediatric nephrologist involved in diagnosing and treating the patient's presentation with nephrotic syndrome. MA was the pediatric resident involved in the care of the patient. JH was the pathologist involved in examining the renal biopsy sample and diagnosing membranous nephropathy. All authors contributed to the article and approved the submitted version.

## Conflict of Interest

The authors declare that the research was conducted in the absence of any commercial or financial relationships that could be construed as a potential conflict of interest.
